# A Study on Differences between Professional Endoscopists and Gastroenterologists in Endoscopic Detection and Standard Pathological Biopsy of Inflammatory Bowel Diseases

**DOI:** 10.1155/2022/7333579

**Published:** 2022-03-26

**Authors:** Dong Yang, Yuqin Li, Haibo Sun, Chuan He, Geng Chen, Zhuo Zhao, Tongyu Tang

**Affiliations:** The First Hospital of Jilin University, Changchun, China

## Abstract

**Objective:**

To assess whether professional endoscopists need additional training on inflammatory bowel disease (IBD) diagnosis.

**Methods:**

This retrospective study was conducted in patients with IBD, including Crohn's disease (CD) and ulcerative colitis (UC), which were diagnosed and treated for the first time in our hospital between January 2005 and December 2020. Doctors including gastroenterologists (group G) and professional endoscopists (group E) participated in the study. The data divided into CD or UC and group G or group E were compared.

**Results:**

Patients with CD exhibited higher rates of terminal ileal lesions, reexamined colonoscopy within 6 months, and intestinal stenosis than patients with UC (*P* < 0.001). The positive endoscopic IBD diagnosis rate was significantly higher in group G than in group E (89.6% vs. 74.0%, *P* < 0.001). In the subgroup analysis for patients with CD, the positive endoscopic IBD diagnosis rate was significantly higher for group G than for group E (81.5% vs. 41.8%, *P* < 0.001). However, the two groups exhibited no significant difference in the subgroup analysis for patients with UC (94.1% vs. 86.5%, *P* = 0.060). Group G exhibited a higher rate of terminal ileal intubation (83.1% vs. 65.3%, *P* < 0.001) and standard pathological biopsy (72.7% vs. 26.0%, *P* < 0.001) than Group E.

**Conclusion:**

Professional endoscopists showed lower rates of terminal ileal intubation, positive endoscopic diagnosis, and standard pathological biopsy than gastroenterologists. Hence, additional training on IBD, particularly on CD, must be provided to professional endoscopists to increase their efficiency for terminal ileal intubation and positive endoscopic diagnosis and to enhance their awareness regarding standard biopsy.

## 1. Introduction

Inflammatory bowel diseases (IBDs) include ulcerative colitis (UC) and Crohn's disease (CD). Although the confirmation of IBD diagnosis in some suspected patients may require a long follow-up period, colonoscopy is still the most crucial diagnostic modality [1]. With increasing recognition of digestive endoscopy, endoscopic diagnosis and treatment have been promoted globally. Digestive endoscopy is currently performed mainly by gastroenterologists and professional endoscopists. However, the diagnosis of IBD, particularly the differentiation between CD and UC, through colonoscopy remains challenging for operators due to the low incidence rate [2] and various manifestations of the disease under endoscopy. The differential diagnosis of UC and CD is crucial mainly because the treatment of the two conditions is different [3, 4], and the dietary guidance provided to patients with CD and UC varies, according to the International Organization for the Study of Inflammatory Bowel Disease [5]. Additionally, taking a biopsy is essential for the confirmation of IBD diagnosis, distinction between UC and CD, and for ruling out the diagnosis of dysplasia and coexistent conditions or complications [6]. In addition, an accurate histopathological assessment is crucial for ensuring the correct diagnosis, subclassification, and management of IBD. The disease activity and disease extent of UC or CD can be assessed on the basis of pathological features [7, 8], which can be useful not only for diagnosis but also for the therapeutic effect. Professional endoscopists are only involved in the endoscopic diagnosis of CD or UC, whereas gastroenterologists perform the endoscopic diagnosis, clinical treatment, and long-term follow-up [3]. Therefore, the present study was conducted to assess differences in the positive diagnostic rate and standardization of endoscopic examination for IBD between the two groups of doctors and to assess whether professional endoscopists require additional training on IBD.

## 2. Materials and Methods

The present retrospective study was conducted in patients with IBD who were diagnosed and treated for the first time in the Department of Gastroenterology at the First Hospital of Jilin University between January 2005 and December 2020. Institutional ethics clearance was obtained for the study. The data of the patients' first colonoscopy examination in our hospital were reviewed. Colonoscopic examinations were performed by gastroenterologists and professional endoscopists, who constituted group G and group E, respectively. Group G was composed of physicians with expertise in IBD who had worked both in the Department of Gastroenterology and the Endoscopy Center and performed the endoscopic diagnosis, clinical treatment, and long-term follow-up of IBD patients. Group E was composed of physicians with expertise in endoscopic diagnosis and treatment who had worked only in the endoscopy center. Because of difficulty in IBD diagnosis, even the final diagnosis may change over time; however, in the present study, we considered the patients' current clinical diagnostic results without dispute and doubt as the diagnostic results. Patients in whom IBD was diagnosed but the final clinical diagnosis of CD or UC was not confirmed; those with a colorectal resection history, those with acute massive hemorrhage of the digestive tract, and those in whom experimental treatment including 5-ASA (5-aminosalicylic acid) and corticosteroids for IBD was administered before the first colonoscopy in our hospital were excluded from the study. However, patients treated with intestinal anti-infective drugs, including antiviral, antibacterial, and antifungal drugs were not excluded from the study.

Baseline data and the first colonoscopy data of the included patients in our hospital were collected and analyzed. Bowel cleanliness was evaluated and divided into four grades, namely, excellent (*E*), good (*G*), fair (*F*), and poor (*P*) [9]. Intestinal stenosis caused by IBD was divided into three types: colorectal, ileocecal valve, and terminal ileal. According to the end point of intubation, the patients were categorized into three groups: no ileocecal intubation, cecal intubation, and terminal ileum intubation. The lesions were categorized into ulceration and erosion lesions, whereas their locations were grouped into four regions, namely, rectum, left colon, right colon, and terminal ileum. According to the distribution characteristics, the lesions were categorized as having continuous and scattered distributions. The diagnosis was considered positive when UC or CD was correctly diagnosed, whereas it was considered negative when either UC was misdiagnosed as CD or CD was misdiagnosed as UC. During the initial endoscopic evaluation, at least two biopsy specimens had to be obtained from each site, namely, ileum, ascending colon, transverse colon, descending colon, sigmoid colon, and rectum, throughout the examined bowel for histological assessment [10, 11]. The standard pathological biopsy was not considered to be performed if these requirements were not met. Cases requiring reexamination within 6 months to confirm the diagnosis were recorded. The data divided into CD or UC and group G or group E were compared.

### 2.1. Statistical Analysis

All statistical analyses were performed using SPSS software (version 20.0 for Windows). Continuous variables with normal distribution are expressed as mean and standard deviation and were compared using Student's *t*-test. Continuous variables with nonnormal distribution are expressed as median (P25, p75) and were compared using Mann–Whitney *U* test. Categorical variables were analyzed using Pearson's chi-square test. A two-tailed *P* value of <0.05 was considered statistically significant.

## 3. Results

**Figure 1:**
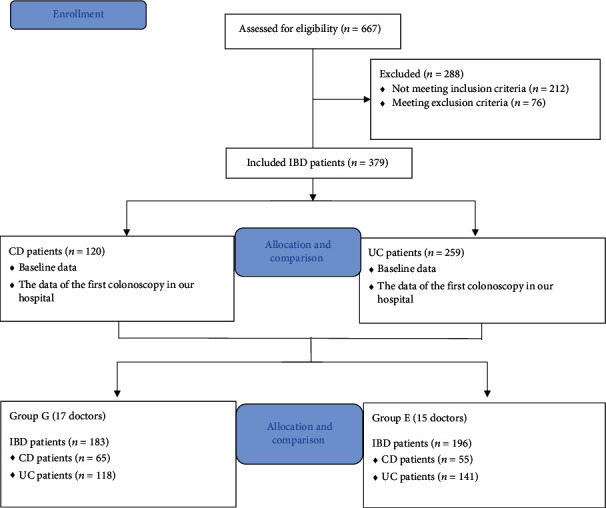
Flow chart detailing the conduct of the study.

From January 2005 to December 2020, 667 patients with IBD, including 198 patients with CD and 469 patients with UC, were treated in the department. Of the total, 212 cases were not diagnosed and treated for the first time in our hospital and thus did not meet the inclusion criteria. Additionally, 43 unclassified IBD cases or with experimental treatment, 26 cases with a history of colectomy, and seven cases accompanied by acute massive hemorrhage of the digestive tract at the initial diagnosis did not meet the inclusion criteria and hence excluded. Finally, 379 patients including 120 patients with CD (31.7%) and 259 patients with UC (68.3%), satisfying the inclusion and exclusion criteria, were included in the study. Colonoscopic examinations were performed by 32 doctors including 17 gastroenterologists and 15 professional endoscopists (Figure 1).

The age of patients with CD was lesser than that of patients with UC (29.5 vs. 43.0, *P* < 0.001). Although both groups exhibited a male predisposition, the percentage of male patients with CD was significantly higher than that of male patients with UC (73.3% vs. 54.1%, *P* < 0.001). The first colonoscopy in our center exhibited no significant difference in the type of colonoscopy, degree of bowel cleanliness, and rate of successful intubation between the two patient groups. The incidence of intestinal stenosis in patients with CD was higher than that in patients with UC (19.2% vs. 1.9%, *P* < 0.001). Patients with CD exhibited higher incidence of ileocecal valve and terminal ileum stenosis than patients with UC. Ulcerations or erosions were observed in most patients with CD during the first colonoscopy, whereas no positive lesion was observed in 9 (7.5%) patients with CD. Further analysis exhibited that eight cases were of small intestinal CD, and one case was of erosion within 6 months. Additionally, erosion was found to be the main manifestation of UC (62.2%). According to the distribution characteristics, except for the cases with no positive lesion under colonoscopy, CD lesions were scattered, with 60% of the cases involving the right colon and the least number (21.7%) of cases involving the rectum. Conversely, UC lesions exhibited continuous distribution, with 91.5% of the cases involving the rectum and only 2.3% of the cases involving the terminal ileum. Positive diagnosis was higher in patients with UC than in patients with CD (90.0% vs. 63.3%, *P* < 0.001). A total of 38 patients underwent colonoscopy reexamination after 6 months because the diagnosis could not be confirmed in these patients. During the 6-month period, either these patients were administered experimental treatment with mesalazine and probiotics or the strategy of waiting and observation were adopted, according to the doctor's guide. Moreover, patients with intestinal infection were administered anti-infection treatment during this period. The percentage of patients with CD who required reexamination within 6 months to confirm the diagnosis was higher than that of patients with UC (17.5% vs. 6.6%, *P* < 0.001) (Table 1). Except for six patients whose periappendiceal part could not be observed after successful intubation due to poor bowel preparation and 33 cases without successful intubation, isolated periappendiceal inflammation was observed in 42 (19.1%) patients with UC, whereas it was not observed in any of the patient with CD.

The median number of patients examined by each doctor was 12.0 (6.0, 18.0). Professional qualifications between group G and group E did not vary significantly. Group G demonstrated a significantly higher percentage of terminal ileum intubation than group E (83.1% vs. 65.3%, *P* < 0.001). However, further subgroup analysis exhibited no significant difference between groups G and E in the percentage of terminal ileum intubation in patients with CD (84.6% vs. 69.1%, *P* = 0.122), whereas the percentage of terminal ileum intubation in patients with UC was significantly higher for group G than for group E (82.2% vs. 63.8%, *P* < 0.001). The positive endoscopic diagnosis rate of IBD and CD for group G was significantly higher than that for group E (89.6% vs. 74.0%, *P* < 0.001 and 81.5% vs. 41.8%, *P* < 0.001, respectively). However, no significant difference was observed between the two groups in terms of the positive endoscopic diagnosis rate of UC (94.1% vs. 86.5%, *P* = 0.060). Group G exhibited a significantly higher rate of standard pathological biopsy under colonoscopy in patients with IBD, CD, and UC than group E (*P* < 0.001) (Table 2).

**Figure 2:**
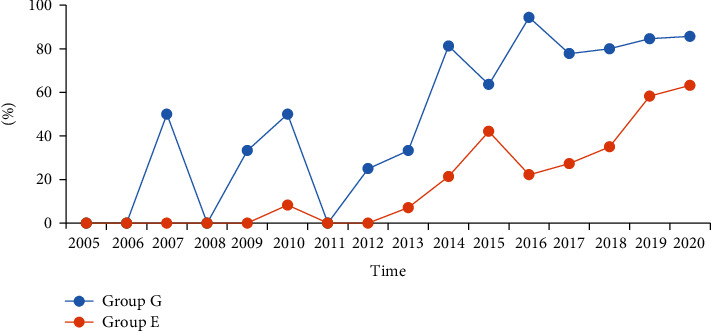
The trend chart of standard pathological biopsy of two groups.

Group G exhibited a significantly higher rate of standard pathological biology under colonoscopy than group E over those years (*P* < 0.001) (Table 3).  Figure 2 depicts the trend chart of the rate of standard pathological biopsy in different years. The percentage of standard pathological biopsy in patients with IBD displayed an increasing trend, especially after 2014.

## 4. Discussion

Clinicians possess relatively less knowledge of IBD screening compared with that of conventional diseases [12] because of the low incidence rate of the disease [13]. Although our center, having a professional team of gastroenterologists for IBD, is the largest endoscopy center in Jilin Province, only 12 (6.0, 18.0) patients have been examined for the first time by each doctor in a span of 16 years. IBD diagnosis may be challenging sometimes, which necessitates a multidisciplinary team and an integrative analysis of data (including clinical, laboratory, endoscopic, imaging, and histologic) for a precise diagnosis. Colonoscopy operators constitute a vital part of the multidisciplinary team and thus should be able to provide useful, standardized, and complete information on endoscopic performance for a comprehensive analysis. Confirming the diagnosis of various IBD manifestations, especially the first endoscopic diagnosis, is challenging for the clinicians performing colonoscopy. Gastroenterologists receive the opportunity to systematically study IBD from diagnosis and treatment to follow-up; however, they do not extensively engage in performing endoscopic diagnosis and treatment compared with professional endoscopists. Therefore, the efficiency of gastroenterologists and professional endoscopists to diagnose IBD should be compared, especially in endoscopic detection and standard pathological biopsy, and attempts should be made to understand whether professional endoscopists should be provided additional training on IBD diagnosis and treatment.

No significant difference was observed in professional qualifications between the two groups (Table 2), and the doctors in both groups had been working together on the same level, confirming that the two groups were ideally matched. The diagnosis of IBD is complex [14–17], and differentiating CD from UC is a challenging task. Approximately 5%–15% of patients with IBD cannot be differentiated on the basis of CD and UC and are labeled as IBD-unclassified (IBD-U) [18, 19]. Colonoscopy is a vital screening tool, and positive diagnosis is a crucial quality control index. In the present study, group G exhibited a higher positive endoscopic diagnosis for IBD and CD than group E (*P* < 0.001). Therefore, professional endoscopists must improve their efficiency for positive endoscopic IBD diagnosis, especially for CD patients. However, no significant difference was observed between the two groups in terms of positive endoscopic diagnosis of UC (*P* > 0.05). This difference might be due to the high incidence of UC in China [13, 20, 21] and diverse CD manifestations under colonoscopy, leading to greater diagnostic difficulty [22–24]. Simultaneously, the percentage of patients who had to be reexamined within 6 months to confirm diagnosis was higher in the CD group (17.5%) than in the UC group (Table 1). The baseline data and colonoscopic manifestations of CD and UC were compared to assess the awareness of colonoscopic features of CD or UC among examiners and examine their ability to differentiate between CD and UC (Table 1). Patients with CD were younger and exhibited a more pronounced male predisposition than those having UC, with colonoscopic manifestations varying from ulceration (43.3%) and erosion (40.8%) to no positive manifestation (7.5%). Additionally, in patients with CD, the lesions were mainly scattered and located in the right colon, whereas in patients with UC, the lesions mainly exhibited a continuous distribution with rectal involvement. Isolated periappendiceal inflammation is the specific manifestation of UC (19.1%) [8, 25, 26]. Thus, although CD or UC can be differentiated according to the typical lesion type and distribution characteristics, the remaining atypical cases must be further differentiated according to other characteristics [27] and pathological biopsy. Interestingly, despite the higher incidence of intestinal stenosis in patients with CD than in patients with UC [28, 29], the successful intubation rate of patients with CD was higher than that of patients with UC; although, the difference was statistically nonsignificant (90.8% vs. 87.3%, *P* > 0.05). The result may be attributed to various reasons. We consider the distribution characteristics of the lesions as one of the reasons. UC lesions were mostly diffused in the rectum and left colon. However, the left colon, particularly the sigmoid colon, was the stress point of intubation. Furthermore, bowel cleanliness of the included patients was poorer [30] than that of other patients in our center [31]. Therefore, when inflammation was particularly severe or loop formation made intubation difficult, colonoscopy could not reach the cecum to avoid the occurrence of associated risks such as bleeding and perforation.

The cecal intubation rate is a vital quality control index in routine colonoscopy [32]. However, the percentage of patients with CD (40.8%) exhibiting terminal ileal lesions was higher than that of patients with UC (2.3%) in the present study (Table 1). Furthermore, the difference in colonoscopic and pathological features between CD ileal involvement and reflux ileitis in UC may be crucial for the differential diagnosis of UC and CD. Therefore, the terminal ileal intubation rate should be considered a vital quality control index in the colonoscopy examination for IBD patients. In all patients with IBD or UC, the terminal ileal intubation rate for group G was significantly higher than that for group E (*P* < 0.001). In patients with CD, the terminal ileal intubation rate for group G was higher than for group E; however, the difference was nonsignificant (84.6% vs. 69.1%, *P* = 0.122), which might be due to the limited sample size of patients with CD (Table 2). Therefore, professional endoscopists must attempt to increase the terminal ileal intubation rate in patients with IBD.

Standard pathological biopsy under colonoscopy is crucial not only for the diagnosis and differential diagnosis but also as a reference for evaluating the treatment affect for CD or UC [6]. The rate of standard pathological biopsy for group G was significantly higher than that for group E in the present study (72.7% vs. 26.0%, *P* < 0.001). Subgroup analysis exhibited that the rate of standard pathological biopsy in both CD and UC groups was significantly higher for group G than for group E (*P* < 0.05). The rate of standard pathological biopsy has been displaying an increasing trend, particularly after 2014, though there still was a significant difference, which may be attributed to the accumulation of endoscopists' experience and understanding of IBD (Table 3 and Figure 2). The findings of this study indicate that professional endoscopists must acquire more information for diagnosis and evaluation of the therapeutic effect by enhancing their awareness of standard pathological biopsy in IBD cases [1, 33].

The two groups of doctors on the same platform in the present retrospective study were ideally matched. However, this study has some limitations. First, the sample size was small. Second, the patients treated with drugs that may cause intestinal inflammation, such as nonsteroidal anti-inflammatory drugs, were not excluded. Third, this study focused on the positive endoscopic IBD diagnosis rates, warranting future studies to evaluate the misdiagnosis rate or over diagnosis of IBD under endoscopy. Moreover, the data in this study are based on a single center; although, the incidence of IBD varies across different regions [2, 20]. Therefore, more large-scale and multicenter prospective studies are required to verify the postulation of the present study.

## 5. Conclusion

Professional endoscopists showed lower rates of terminal ileal intubation, positive endoscopic diagnosis, and standard pathological biopsy than gastroenterologists. Hence, they must be provided additional training on IBD, particularly on CD, so that their efficiency for terminal ileal intubation and positive endoscopic diagnosis can be increased, and their awareness regarding standard biopsy can be enhanced.

## Figures and Tables

**Table 1:** Comparison between the baseline and colonoscopy data of patients with CD and those with UC.

	CD (*n* = 120)	UC (*n* = 259)	Statistic	*P* value
Sex			*χ* ^2^ = 12.717	<0.001
Male	88 (73.3)	140 (54.1)		
Female	32 (26.7)	119 (45.9)		
Age (years)	29.5 (22.0,38.0)	43.0 (31.0, 56.0)	*Z* = 22258.500	<0.001
Colonoscopy type			*χ* ^2^ = 2.673	0.102
Unsedated colonoscopy	15 (12.5)	50 (19.3)		
Sedated colonoscopy	105 (87.5)	209 (80.7)		
Bowel preparation			*χ* ^2^ = 7.621	0.055
Excellent	3 (2.5)	5 (1.9)		
Good	33 (27.5)	46 (17.8)		
Fair	80 (66.7)	186 (71.8)		
Poor	4 (3.3)	22 (8.5)		
Intestinal stenosis			*χ* ^2^ = 40.310	<0.001
Colorectum	10 (8.3)	5 (1.9)		
Ileocecal valve	8 (6.7)	0		
Terminal ileum	5 (4.2)	0		
No	97 (80.8)	254 (98.1)		
End point of intubation			*χ* ^2^ = 1.382	0.502
No ileocecal intubation	11 (9.2)	33 (12.7)		
Cecal intubation	16 (13.3)	39 (15.1)		
Terminal ileum intubation	93 (77.5)	187 (72.2)		
Type of lesions			*χ* ^2^ = 112.317	<0.001
Ulcer	52 (43.3)	11 (4.2)		
Erosion	49 (40.8)	161 (62.2)		
Ulcer and erosion	10 (8.3)	87 (33.6)		
No	9 (7.5)	0		
Location of lesions			*χ* ^2^ = 176.844	<0.001
Rectum	26 (21.7)	237 (91.5)		
Left colon	49 (40.8)	202 (80.0)		
Right colon	72 (60.0)	96 (37.1)		
Terminal ileum	49 (40.8)	6 (2.3)		
Distribution of lesions			*χ* ^2^ = 124.824	<0.001
Continuous distribution	18 (16.2)	203 (78.4)		
Scattered distribution	93 (83.8)	56 (21.6)		
Positive diagnosis			*χ* ^2^ = 38.614	<0.001
Yes	76 (63.3)	233 (90.0)		
No	44 (36.7)	26 (10.0)		
Reexamined colonoscopy in six months			*χ* ^2^ = 10.872	0.001
Yes	21 (17.5)	17 (6.6)		
No	99 (82.5)	242 (93.4)		

CD: Crohn's disease; UC: ulcerative colitis.

**Table 2:** Comparison of professional qualifications and patient outcomes between group G and group E.

	Group G	Group E	Statistic	*P*
Professional qualifications			*χ* ^2^ = 0.532	0.895
Primary	3 (17.6)	2 (13.3)		
Intermediate	9 (52.9)	7 (46.7)		
Senior	5 (29.4)	6 (40.0)		
Terminal point of intubation			*χ* ^2^ = 19.926	<0.001
No ileocecal intubation	19 (10.4)	25 (12.8)		
Cecal intubation	12 (6.6)	43 (21.9)		
Terminal ileum intubation	152 (83.1)	128 (65.3)		
CD			*χ* ^2^ = 4.121	0.122
No ileocecal intubation	4 (6.2)	7 (12.7)		
Cecal intubation	6 (9.2)	10 (18.2)		
Terminal ileum intubation	55 (84.6)	38 (69.1)		
UC			*χ* ^2^ = 17.321	<0.001
No ileocecal intubation	15 (12.7)	18 (12.8)		
Cecal intubation	6 (5.1)	33 (23.4)		
Terminal ileum intubation	97 (82.2)	90 (63.8)		
Positive diagnosis of IBD			*χ* ^2^ = 15.369	<0.001
Yes	164 (89.6)	145 (74.0)		
No	19 (10.4)	51 (26.0)		
CD			*χ* ^2^ = 20.240	<0.001
Yes	53 (81.5)	23 (41.8)		
No	12 (18.5)	32 (58.2)		
UC			*χ* ^2^ = 4.047	0.060
Yes	111 (94.1)	122 (86.5)		
No	7 (5.9)	19 (13.5)		
Standard pathological biopsy			*χ* ^2^ = 82.477	<0.001
Yes	133 (72.7)	51 (26.0)		
No	50 (27.3)	145 (74.0)		
CD			*χ* ^2^ = 36.226	<0.001
Yes	50 (76.9)	12 (21.8)		
No	15 (23.1)	43 (78.2)		
UC			*χ* ^2^ = 46.963	<0.001
Yes	83 (70.3)	39 (27.7)		
No	35 (29.7)	102 (72.3)		

CD: Crohn's disease; UC: ulcerative colitis; IBD: inflammatory bowel disease.

**Table 3:** Comparison of standard pathological biology between group G and group E.

Time	The percentage of standard pathological biopsy in group G (%)	The percentage of standard pathological biopsy in group E (%)	Statistic	*P*
2005	0	0		
2006	0	0		
2007	50	0		
2008	0	0		
2009	33.3	0		
2010	50	8.3		
2011	0	0		
2012	25	0		
2013	33.3	7.1		
2014	81.3	21.4		
2015	63.6	42.1		
2016	94.4	22.2		
2017	77.8	27.3		
2018	80	35		
2019	84.6	58.3		
2020	85.7	63.2		
			*χ* ^2^ = 82.477	<0.001

## Data Availability

Data is available on request. The data underlying this article will be shared on reasonable request to the corresponding author.

## References

[1] Leighton J. A., Shen B., Baron T. H. (2006). ASGE guideline: endoscopy in the diagnosis and treatment of inflammatory bowel disease. *Gastrointestinal Endoscopy*.

[2] Yu Q., Zhu C., Feng S. (2021). Economic burden and health care access for patients with inflammatory bowel diseases in China: web-based survey study. *Journal of Medical Internet Research*.

[3] Amiot A., Bouguen G., Bonnaud G. (2021). Clinical guidelines for the management of inflammatory bowel disease: update of a French national consensus. *Digestive and Liver Disease*.

[4] Ponsioen C. Y., de Groof E. J., Eshuis E. J. (2017). Laparoscopic ileocaecal resection versus infliximab for terminal ileitis in Crohn's disease: a randomised controlled, open-label, multicentre trial. *The Lancet Gastroenterology & Hepatology*.

[5] Levine A., Rhodes J. M., Lindsay J. O. (2020). Dietary guidance from the International organization for the study of inflammatory bowel diseases. *Clinical Gastroenterology and Hepatology*.

[6] Feakins R. M., British Society of Gastroenterology (2013). Inflammatory bowel disease biopsies: updated British Society of Gastroenterology reporting guidelines. *Journal of Clinical Pathology*.

[7] Hanauer S. B., Sandborn W. J. (2007). European evidence-based consensus on the diagnosis and management of Crohn's disease. *Gut*.

[8] Stange E. F., Travis S. P., Vermeire S. (2008). European evidence-based consensus on the diagnosis and management of ulcerative colitis: definitions and diagnosis. *Journal of Crohn's & Colitis*.

[9] Rex D. K., Petrini J. L., Baron T. H. (2006). Quality indicators for colonoscopy. *The American Journal of Gastroenterology*.

[10] Sandhu B. K., Fell J. M., Beattie R. M., Mitton S. G., Wilson D. C., Jenkins H. (2010). Guidelines for the management of inflammatory bowel disease in children in the United Kingdom. *Journal of Pediatric Gastroenterology and Nutrition*.

[11] Jenkins D., Balsitis M., Gallivan S. (1997). Guidelines for the initial biopsy diagnosis of suspected chronic idiopathic inflammatory bowel disease. The British Society of Gastroenterology Initiative. *Journal of Clinical Pathology*.

[12] Gupta S., Lieberman D., Anderson J. C. (2020). Recommendations for follow-up after colonoscopy and polypectomy: a consensus update by the US multi-society task force on colorectal cancer. *Gastrointestinal Endoscopy*.

[13] Yang H., Li Y., Wu W. (2014). The incidence of inflammatory bowel disease in northern China: a prospective population-based study. *PLoS One*.

[14] Panes J., Bouzas R., Chaparro M. (2011). Systematic review: the use of ultrasonography, computed tomography and magnetic resonance imaging for the diagnosis, assessment of activity and abdominal complications of Crohn’s disease. *Alimentary Pharmacology & Therapeutics*.

[15] Horsthuis K., Bipat S., Stokkers P. C., Stoker J. (2009). Magnetic resonance imaging for evaluation of disease activity in Crohn's disease: a systematic review. *European Radiology*.

[16] Yu H., Liu Y., Wang Y., Peng L., Li A., Zhang Y. (2012). Clinical, endoscopic and histological differentiations between Crohn's disease and intestinal tuberculosis. *Digestion*.

[17] Axelrad J. E., Joelson A., Green P. H. R. (2018). Enteric infections are common in patients with flares of inflammatory bowel disease. *The American Journal of Gastroenterology*.

[18] Tremaine W. J. (2007). Review article: indeterminate colitis – definition, diagnosis and management. *Alimentary Pharmacology & Therapeutics*.

[19] Guindi M., Riddell R. H. (2004). Indeterminate colitis. *Journal of Clinical Pathology*.

[20] Zeng Z., Zhu Z., Yang Y. (2013). Incidence and clinical characteristics of inflammatory bowel disease in a developed region of Guangdong Province, China: a prospective population-based study. *Journal of Gastroenterology and Hepatology*.

[21] Wang Y., Ouyang Q., APDW 2004 Chinese IBD Working Group (2007). Ulcerative colitis in China: retrospective analysis of 3100 hospitalized patients. *Journal of Gastroenterology and Hepatology*.

[22] Lennard-Jones J. E. (1989). Classification of inflammatory bowel disease. *Scandinavian Journal of Gastroenterology*.

[23] Lamb C. A., Kennedy N. A., Raine T. (2019). British Society of Gastroenterology consensus guidelines on the management of inflammatory bowel disease in adults. *Gut*.

[24] Samuel S., Bruining D. H., Loftus E. V. (2012). Endoscopic skipping of the distal terminal ileum in Crohn's disease can lead to negative results from ileocolonoscopy. *Clinical Gastroenterology and Hepatology*.

[25] Matsumoto T., Nakamura S., Shimizu M., Iida M. (2002). Significance of appendiceal involvement in patients with ulcerative colitis. *Gastrointestinal Endoscopy*.

[26] Byeon J. S., Yang S. K., Myung S. J. (2005). Clinical course of distal ulcerative colitis in relation to appendiceal orifice inflammation status. *Inflammatory Bowel Diseases*.

[27] Park S. H., Yang S. K., Park S. K. (2014). Atypical distribution of inflammation in newly diagnosed ulcerative colitis is not rare. *Canadian Journal of Gastroenterology & Hepatology*.

[28] Rieder F., Zimmermann E. M., Remzi F. H., Sandborn W. J. (2013). Crohn's disease complicated by strictures: a systematic review. *Gut*.

[29] Navaneethan U., Lourdusamy V., Njei B., Shen B. (2016). Endoscopic balloon dilation in the management of strictures in Crohn's disease: a systematic review and meta-analysis of non-randomized trials. *Surgical Endoscopy*.

[30] Froehlich F., Wietlisbach V., Gonvers J. J., Burnand B., Vader J. P. (2005). Impact of colonic cleansing on quality and diagnostic yield of colonoscopy: the European panel of appropriateness of gastrointestinal endoscopy European multicenter study. *Gastrointestinal Endoscopy*.

[31] Yang D., Tao K., Chen G., Zhang L., He Q., Xu H. (2020). Randomized controlled trial of polyethylene glycol versus oral sodium phosphate for bowel preparation in unsedated colonoscopy. *Gastroenterology Research and Practice*.

[32] Choi J. H., Cha J. M., Yoon J. Y., Kwak M. S., Jeon J. W., Shin H. P. (2019). The current capacity and quality of colonoscopy in Korea. *Intestinal Research*.

[33] Geboes K., Ectors N., D'Haens G., Rutgeerts P. (1998). Is ileoscopy with biopsy worthwhile in patients presenting with symptoms of inflammatory bowel disease?. *The American Journal of Gastroenterology*.

